# Psychometric Properties of the Strengths and Difficulties Questionnaire-Dysregulation Profile in Italian Early Adolescents

**DOI:** 10.3390/children8121123

**Published:** 2021-12-03

**Authors:** Valentina Levantini, Eleonora Cei, Gennarina Pirri, Pietro Muratori

**Affiliations:** 1 IRCCS Fondazione Stella Maris, 56128 Pisa, Italy; ec.eleonoracei@gmail.com (E.C.); pietro.muratori@fsm.unipi.it (P.M.); 2 Centro Salute Trieste, 34100 Trieste, Italy; jennypirri@gmail.com

**Keywords:** hyperactivity, conduct problems, behavior, prosocial, school

## Abstract

Emotional dysregulation is of great cause for concern because it is associated with severe outcomes. Currently, the identification of youths with signs of emotional and behavioral dysregulation is obtained through the assessment of a Dysregulation Profile (DP), including the Strengths and Difficulties Questionnaire-DP (SDQ-DP). Despite its increasingly frequent use in research, studies exploring the SDQ-DP properties are still limited, and no study with Italian samples is currently available. The current study aimed to explore the psychometric properties of the SDQ-DP parent-report and its association with difficulties in the school context in a sample of 332 Italian early adolescents. Results showed that the SDQ-DP parent-report is a single-factor measure with good internal consistency. Also, in both males and females, the SDQ-DP parent-report was associated with higher teacher-reported Internalizing (e.g., anxious symptoms) and Externalizing Problems (e.g., hyperactivity, conduct problems) and lower Prosocial Behavior. This study added further evidence about the utility of the SDQ-DP in the assessment, prevention, and treatment of emotional dysregulation.

## 1. Introduction

A considerable number of early adolescents present a complex picture of co-occurring emotional, behavioral, and cognitive dysregulation, causing significant adjustment problems [1,2,3]. Usually, the identification of emotional dysregulation in youths is achieved thanks to the assessment of a Dysregulation Profile (DP), which is developed to measure psychopathological symptoms sustained by deficits in self-regulation of emotions and behaviors. 

This index is associated with self-regulatory problems in multiple domains and represents a risk marker for psychiatric problems and youths’ poor adjustment [4]. The DP, based on the Child Behavior Checklist (CBCL) [5], has emerged as a reliable and valid measure of this complex phenotype of dysregulation in samples from community and clinical contexts [6,7,8]. However, the CBCL is a long and expensive tool, not easy to use in screening studies and primary care settings.

A specific DP score based on the Strengths and Difficulties Questionnaire (SDQ) [9] has also been validated [10,11,12]. The SDQ is a 25-item open-access measure, which assesses Prosocial Behavior, Hyperactivity–Inattention, Emotional Symptoms, Conduct Problems, and Peer Problems. Its brevity makes it less time-consuming than other measures without compromising its reliability [13,14] and it is more feasible for quick screening in health care settings [15,16]. The SDQ is available in several languages, including Italian, and, in Italy, it is frequently used in studies involving both clinical [17] and community samples [18]. Both the Italian parent-report and teacher-report forms have been thoroughly validated [19,20]. Overall, they showed similar psychometric properties to Goodman’s original SDQ [9]. 

The SDQ-DP, as defined by Holtmann et al. [12], represents the combination of SDQ items, which best discriminates youths with and without the CBCL-DP. The SDQ-DP is computed by adding up the scores of five items of the SDQ: one from the Hyperactivity–Inattention subscale (item 2: “Restless, overactive, cannot stay still”); two from the Conduct Problems scale (items 12: “Often fight with other youth or bullies them”; item 22: “Steal from home, school, or elsewhere”); and two from the Emotional Symptoms scale (items 8: “Many worries or often seems worried”; item 13: “Often unhappy, depressed, tearful”). The SDQ-DP showed optimal sensitivity and specificity (respectively, 94.60% and 80%) and appeared to be highly correlated with the CBCL-DP score. This suggests that the SDQ-DP well reflects the signs of dysregulation described by the CBCL-DP, despite its low number of items. Regarding the SDQ-DP internal consistency, studies have found low to moderate reliability (Cronbach’s alphas: 0.52–0.65) [11,12]. However, it is important to highlight that some internal consistency measures, i.e., Cronbach’s alphas, may be affected by short-item scales. Despite its increasing popularity, studies evaluating the psychometric properties of the SDQ-DP are still lacking, and no previous studies investigated the SDQ-DP properties in Italian populations. 

Furthermore, early adolescence is a period of transition with significant biological, physical, mental, and social changes and challenges; thus, the evaluation of a screening measure of dysregulation might be particularly important for early adolescents to identify youths at risk for developing psychopathological symptoms [21]. In this regard, previous studies have shown that the SDQ-DP is associated with emotional and behavioral problems, greater severity of symptomatology, and poorer psychosocial functioning—e.g., [12,22]. Interestingly, there is also evidence suggesting gender differences in both prevalence and severity regarding the SDQ-DP scores. However, the findings showed some inconsistencies. Some studies showed that boys reported significantly higher SDQ-DP scores and were more affected than girls and that males and females might follow partially different trajectories [11,23]. In contrast, other studies found no gender differences [10,22]. 

The SDQ-DP, as described above, has been validated to screen the DP in a clinical setting [12]. The SDQ-DP was developed to mirror the CBCL-DP, a parent-report measure, and it comes as a briefer and easier to use tool. Therefore, the aim of the current study is to examine the factor structure and the reliability of the SDQ-DP parent-report in a sample of Italian students. In line with Holtmann et al. [12], this study examined the ability of the SDQ-DP parent-report scores to capture the presence of emotional and behavioral difficulties in youths (assessed with the SDQ teacher-report). Finally, since data on this matter are currently mixed, we tested whether gender moderated the association between the parent-reported SDQ-DP scores and the abovementioned outcomes—see for instance [18,24].

## 2. Materials and Methods

### 2.1. Participants and Procedure

The sample was collected in schools located in the northeast of Italy. It included 332 (49.70% females) middle school Italian students aged 10–14 years old (mean age = 11.95, *SD* = 0.64). Eighty-six students (25.90%) were 6th graders, while 246 (74.10%) were 7th graders.

All the students’ parents and teachers were asked to complete the SDQ. Before filling in the questionnaires, all parents, students, and teachers signed a written informed consent and agreed to participate in the study. The procedures were in accordance with the ethical standards of the institutional research committee and with the 1964 Helsinki declaration, and its later amendments or comparable ethical standards. This study was approved by the Institutional Review Board of Istituto Comprensivo Pascoli (PTOF, prot. 13, 13 October 2020).

### 2.2. Measures

Strengths and Difficulties Questionnaire: The SDQ [9] is a 25-item questionnaire that assesses youth emotional and behavioral difficulties and strengths. It is available in different formats, including parent-report and teacher-report versions. All the answers are provided on a 3-point Likert scale (0 = not true, 1 = somewhat true, 2 = certainly true). The SDQ involves four subscales evaluating youths’ difficulties, namely Hyperactivity–Inattention, Emotional Symptoms, Conduct Problems, Peer Problems, and one subscale assessing the youths’ strengths, Prosocial Behavior. Each subscale includes five items, and its score ranges from 0 to 10. Higher scores suggest a higher degree of difficulties, except for the Prosocial Behavior score. The subscale’s scores can be grouped to get an Internalizing Problems (Emotional Symptoms + Peer Problems) and an Externalizing Problems (Hyperactivity–Inattention + Conduct Problems) score. These scores range from 0 to 20, and higher scores indicate higher Internalizing or Externalizing Problems levels. The use of these composite scores is recommended, especially for community samples [25]. For the current study, we used the Italian version of the SDQ parent-report [19] and teacher-report [20]. The SDQ-DP parent-report has been computed following Holtmann et al.’s [12] definition.

School grades: School grades were derived from class assignments, and scores ranged from 0 to 10 (with ten being the best mark). Teachers reported the mean grade of all school subjects for each student. 

### 2.3. Statistical Analysis

The statistical tests were run on IBM SPSS Statistics for Windows, Version 26.0 (Armonk, NY, USA) unless otherwise stated. The percentage of missing values across all the variables varied between 0% and 14.7%. Overall, 8.07% of the values were incomplete. Missing data were imputed using multiple imputations (*n* = 10). Incomplete variables were imputed under fully conditional specification, using the default setting “Impute Missing Data Values (Multiple Imputation)” available on SPSS. 

As a preliminary analysis, we ran Confirmatory Factor Analysis (CFA) separately for males and females. Since males and females showed similar factor loadings (all factor loadings ≥ 0.50), and the goodness-of-fit indices were excellent for both groups, we decided to run a CFA for the whole sample. The CFA was performed in IBM SPSS Amos, Version 26.0, using the maximum likelihood estimator. We assessed the model’s goodness-of-fit using different indices, including a chi-square (*χ*^2^) statistic, a comparative fit index (CFI), a Tucker–Lewis index (TLI), and a root mean square error of approximation (RMSEA). We interpreted TLI and CFI values ≥ 0.90 as acceptable and ≥0.95 as excellent, and RMSEA values ≤ 0.08 as acceptable and ≤0.06 as excellent [26]. We tested a single-factor model for the SDQ-DP.

The SDQ-DP parent-report internal consistency has been assessed with Cronbach’s alpha and the mean inter-item correlations (MICs). The latter index is particularly relevant for short item scales; ideally, the MICs should not be lower than 0.15 or higher than 0.50. Low MICs suggest that the items are not well correlated and do not measure the same construct. When MICs are too high, the items are possibly too close and redundant. 

To identify the prevalence of students with affective and behavioral dysregulation, we divided the participants into those who met the SDQ-DP criteria (SDQ-DP score ≥ 5) and those who did not (SDQ-DP score < 5), following Holtmann et al.’s [12] definition.

We then used zero-order correlations to test whether the parent-reported SDQ-DP was associated with students’ age, gender, school grades, teacher-rated Internalizing and Externalizing Problems, and Prosocial Behavior.

Finally, we tested whether gender moderated the association between parent-reported SDQ-DP and school grades, teacher-rated Internalizing Problems and Externalizing Problems, and Prosocial Behavior with a series of moderation models. All the continuous variables that defined products were mean-centered before the analysis. 

## 3. Results

### 3.1. Psychometric Properties and Prevalence

In the whole sample, the SDQ-DP had a mean score of 2.07 (*SD* = 2.32). Males reported a mean score of 2.57 (*SD* = 2.49), while females had one of 1.56 (*SD* = 2.02), showing that females had significantly lower levels of affective and behavioral dysregulation (*t* = −4.06, *p* < 0.001, C.I. [−1.50, −0.52]). Overall, 14.75% of the students (*n* = 49; 34 males and 15 females) met the criteria for the SDQ-DP parent-report.

**Figure 1:**
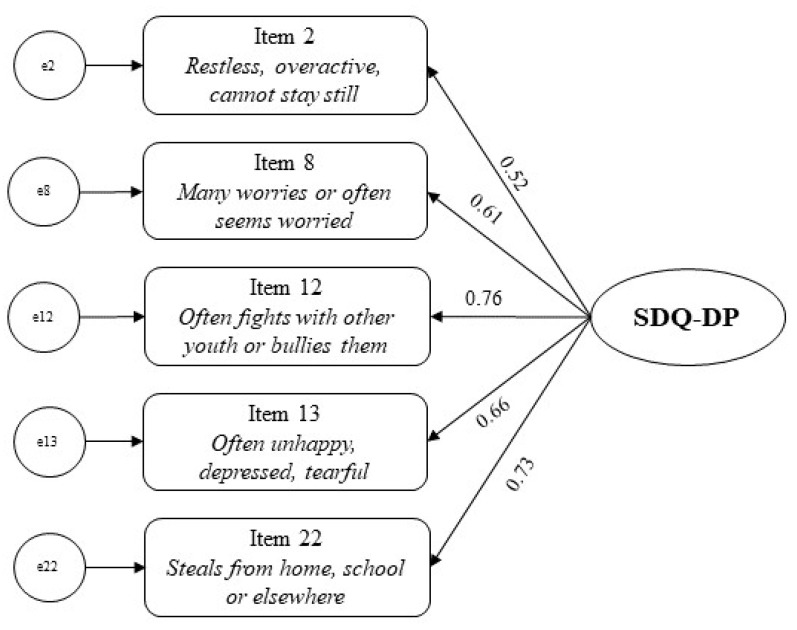
Factor loadings for the SDQ-DP parent-report.

The CFA showed that a single-factor model provided adequate fit to the data (*χ*^2^ (5) = 10.042, *p* = 0.074; CFI = 0.988; TLI = 0.977; RMSEA = 0.055). All the factor loadings were ≥0.50 (see Figure 1). 

The parent-reported SDQ-DP had a Cronbach’s alpha of 0.76, and the MICs were 0.43, suggesting good internal consistency.

### 3.2. Zero-Order Correlations

Bivariate correlations (see Table 1) showed that male gender was associated with higher affective and behavioral dysregulation; the parent-reported SDQ-DP was positively associated with teacher-reported Internalizing Problems (*r* = 0.438, *p* < 0.001, C.I. [0.337, 0.537]) and Externalizing Problems (*r* = 0.429, *p* < 0.001, C.I. [0.320, 0.525]), and negatively associated with teacher-reported Prosocial Behavior (*r* = −0.325, *p* < 0.001, C.I. [−0.425, −0.230]). The SDQ-DP was not related to students’ age and school grades.

### 3.3. Moderation Models

The moderation models showed that gender did not moderate the associations between the SDQ-DP parent-report and teacher-reported Internalizing (*b* = 0.126, *p* = 0.429, C.I. [−0.188, 0.441]) and Externalizing Problems (*b* = 0.281, *p* = 0.145, C.I. [−0.098, 0.660]), Prosocial Behavior (*b* = −0.089, *p* = 0.390, C.I. [−0.293, 0.115]), nor school grades (*b* = 0.047, *p* = 0.211, C.I. [−0.027, 0.120]).

Further analyses revealed that, in both males and females, the SDQ-DP parent-report was positively associated with teacher-reported Internalizing (males: *b* = 0.71, *p* < 0.001, C.I. [0.504, 0.906]; females: *b* = 0.58, *p* < 0.001, C.I. [0.337, 0.821]) and Externalizing Problems (males: *b* = 0.81, *p* < 0.001, C.I. [0.530, 1.098]; females: *b* = 0.53, *p* < 0.001, C.I. [0.307, 0.759]), and negatively associated with Prosocial Behavior (males: *b* = −0.30, *p* < 0.001, C.I. [−0.429, −0.172]; females: *b* = −0.21, *p* < 0.001, C.I. [−0.371, −0.052]).

## 4. Discussion

In recent years, there has been an increasing interest in exploring youths’ emotional dysregulation. This concern is justified because a combination of emotional and behavioral dysregulation in youths is associated with severe outcomes, including internalizing and externalizing problems, greater risk for psychopathology, and difficulties in the school context (i.e., poor academic performance and school adjustment) [22,27].

The growing awareness about the long-term consequences that emotional dysregulation can yield has made it necessary for the development of new tools to measure it reliably and identify those youths at greater risk for adverse outcomes.

The first tool to be used for assessing emotional dysregulation was the CBCL-DP. More recently, an equivalent DP based on the items of the SDQ parent-report has been developed [12]. Both dysregulation profiles similarly evaluate signs of dysregulation at the emotional, cognitive, and behavioral levels [12]. The SDQ has the benefit of being an open-access questionnaire composed of a reduced number of items, though as reliable as other longer scales [13]. These characteristics make the SDQ widely accessible and suitable for settings characterized by tight time constraints. 

However, it is important to highlight that studies evaluating the SDQ-DP reliability are still limited, and, currently, no studies have explored its psychometric properties in Italian samples. The current study fits within this field of research and aims to test the factor structure and internal consistency of the SDQ-DP parent-report in a community sample of Italian early adolescents. 

Results showed that, in our sample, the SDQ-DP parent-report reliably assessed a single construct. The goodness-of-fit indices (i.e., *χ*^2^, CFI, TLI, RMSEA) indicated that a single-factor model fit our data. Besides, in contrast to previous studies [11,12], in our sample the SDQ-DP parent-report showed acceptable internal consistency assessed with the Cronbach’s alpha (α = 0.76) and the MICs. The latter measure is particularly relevant for short-item scales and, in our sample, showed that the five items composing the SDQ-DP are correlated with each other, but not too much—suggesting they measure the same construct without being redundant. This is a particularly relevant finding since some previous studies found poor reliability for the SDQ-DP [11,12].

Moreover, our findings showed that the parent-reported SDQ-DP scores were associated with difficulties in the school context. Specifically, higher SDQ-DP scores were associated with higher teacher-reported Internalizing and Externalizing Problems and lower Prosocial Behavior. These results are consistent with previous studies, showing that the SDQ-DP is associated with adverse outcomes in both community and clinical samples of youths—e.g., [8]. However, similarly to a previous study [22], the SDQ-DP parent-report was unrelated to the students’ academic performance. 

Some previous studies have suggested that gender differences might be present with regards to the levels of the SDQ-DP. Consistent with Deutz et al. [11], we found that boys reported significantly higher levels of DP assessed with the SDQ parent-report. However, other studies did not find gender differences [10,22], suggesting that further research is needed to shed light on this topic. We also tested whether gender moderated the relationship between parent-reported SDQ-DP scores and the abovementioned outcomes. Although females reported lower SDQ-DP scores, the presence of emotional and behavioral dysregulation in both males and females was equally associated with difficulties in the school context. Boys and girls might show signs of maladjustment in different ways, primarily due to differences in several factors, including biological factors, personality traits, and gender roles. The different presentation of the boys’ and girls’ emotional and behavioral dysregulation, along with different expectations from parents and teachers, might interfere with the identification of important warning signs, especially in girls—see for instance [28,29]. Our results highlight the importance of not underestimating the severe outcomes that might accompany even—only seemingly—less relevant DP levels. 

The results of the current study need to be interpreted in light of some limitations. Our sample included only students within a limited age range; therefore, future studies should explore the SDQ-DP psychometric properties in wider community samples, as well as in samples of clinic-referred youths. Also, we assessed difficulties in the school context only with the SDQ teacher-report. Future studies would benefit from including different external criteria. Future studies should try to individuate the clinical correlates and longitudinal stability of SDQ-DP. Moreover, we did not collect information about the students’ socioeconomic status and their parents’ educational levels. Also, we did not test the role of socio-demographic variables (e.g., age, ethnicity, socioeconomic status, parental education level) in moderating the association between the SDQ-DP parent-report and the studied outcomes. However, previous studies showed that the SDQ-DP was not associated with differences in several socio-demographic characteristics—see for instance [10]. Finally, it is important to mention that an adjunctive SDQ-DP has been proposed by Deutz et al. [11]. This SDQ-DP is computed using all the items from the Conduct Problems, Hyperactivity–Inattention, and Emotional Symptoms subscales, for a total of 15 items. Future studies should also explore the psychometric properties of the 15-item SDQ-DP and its associations with external measures.

Overall, our results showed that the SDQ-DP parent-report is a reliable measure of emotional and behavioral dysregulation in a sample of Italian early adolescents. We also found that more than 14% of the students met the criteria for the SDQ-DP, whose scores were associated with more significant difficulties in the school context. These results highlight the importance of finding proper tools to identify youths at greater risk for severe outcomes, and promptly address their difficulties and prevent them from heading towards the most unfavorable pathways [30]. At the same time, the SDQ-DP scores could also help guide intervention programs in clinical settings too [31]. These implications might be of particular relevance for early adolescence since youths must face several changes during this phase, and a high percentage of them develop Internalizing and Externalizing Problems [32]. 

## Figures and Tables

**Table 1:** Zero-order correlations.

		1	2	3	4	5	6	7
1.	SDQ-DP (Parent)	1						
2.	Gender	0.218 **	1					
3.	Age	0.037	0.017	1				
4.	School Grades	−0.074	−0.098	−0.043	1			
5.	Internalizing (Teacher)	0.438 **	0.152 **	0.081	−0.066	1		
6.	Externalizing (Teacher)	0.429 **	0.430 **	0.132 *	−0.098	0.424 **	1	
7.	Prosocial (Teacher)	−0.325 **	−0.309 **	−0.081	0.133 *	−0.473 **	−0.514 **	1
*Mean*		2.07	-	11.95	7.62	3.55	4.52	6.89
*SD*		2.32	-	0.64	0.76	3.58	4.64	2.28

Note. SDQ-DP: Strengths and Difficulties Questionnaire-Dysregulation Profile. Gender was dummy coded as 1 = male and 0 = female. * *p* < 0. 05, ** *p* < 0.01.

## Data Availability

The data are available from the corresponding author upon reasonable request.

## References

[1] Smorti M., Milone A., Gonzalez J.G., Rosati G.V. (2019). Adolescent selfie: An Italian Society of Paediatrics survey of the lifestyle of teenagers. Ital. J. Pediatr..

[2] Muratori P., Paciello M., Castro E., Levantini V., Masi G., Milone A., Senese V.P., Pisano S., Catone G. (2021). At-risk early adolescents profiles in the community: A cluster analysis using the strengths and difficulties questionnaire. Psychiatry Res..

[3] Vugteveen J., de Bildt A., Hartman C.A., Reijneveld S.A., Timmerman M.E. (2020). The combined self- and parent-rated SDQ score profile predicts care use and psychiatric diagnoses. Eur. Child Adolesc. Psychiatry.

[4] Paulus F.W., Ohmann S., Möhler E., Plener P., Popow C. (2021). Emotional Dysregulation in Children and Adolescents With Psychiatric Disorders. A Narrative Review. Front. Psychiatry.

[5] Achenbach T.M., Rescorla L.A. (2014). The Achenbach System of Empirically Based Assessment (ASEBA) for Ages 1.5 to 18 Years. The Use of Psychological Testing for Treatment Planning and Outcomes Assessment.

[6] Althoff R.R., Rettew D.C., Ayer L.A., Hudziak J.J. (2010). Cross-informant agreement of the Dysregulation Profile of the Child Behavior Checklist. Psychiatry Res..

[7] Masi G., Pisano S., Milone A., Muratori P. (2015). Child behavior checklist dysregulation profile in children with disruptive behavior disorders: A longitudinal study. J. Affect. Disord..

[8] Deutz M.H.F., Geeraerts S.B., van Baar A., Deković M., Prinzie P. (2016). The Dysregulation Profile in middle childhood and adolescence across reporters: Factor structure, measurement invariance, and links with self-harm and suicidal ideation. Eur. Child Adolesc. Psychiatry.

[9] Goodman R. (1997). The Strengths and Difficulties Questionnaire: A Research Note. J. Child Psychol. Psychiatry.

[10] Carballo J.J., Serrano-Drozdowskyj E., Nieto R.G., De Neira-Hernando M.D., Pérez-Fominaya M., Molina-Pizarro C.A., De Leon-Martinez V., Baca-García E. (2014). Prevalence and Correlates of Psychopathology in Children and Adolescents Evaluated with the Strengths and Difficulties Questionnaire Dysregulation Profile in a Clinical Setting. Psychopathology.

[11] Deutz M.H.F., Shi Q., Vossen H.G.M., Huijding J., Prinzie P., Deković M., Van Baar A.L., Woltering S. (2018). Evaluation of the Strengths and Difficulties Questionnaire-Dysregulation Profile (SDQ-DP). Psychol. Assess..

[12] Holtmann M., Becker A., Banaschewski T., Rothenberger A., Roessner V. (2011). Psychometric Validity of the Strengths and Difficulties Questionnaire-Dysregulation Profile. Psychopathology.

[13] Goodman R., Scott S. (1999). Comparing the Strengths and Difficulties Questionnaire and the Child Behavior Checklist: Is Small Beautiful?. J. Abnorm. Child Psychol..

[14] Klasen H., Woerner W., Wolke D., Meyer R., Overmeyer S., Kaschnitz W., Rothenberger A., Goodman R. (2000). Comparing the German Versions of the Strengths and Difficulties Questionnaire (SDQ-Deu) and the Child Behavior Checklist. Eur. Child Adolesc. Psychiatry.

[15] Barrios-Fernandez S., Gozalo M., Amado-Fuentes M., Carlos-Vivas J., Garcia-Gomez A. (2021). A Short Version of the EFECO Online Questionnaire for the Assessment of Executive Functions in School-Age Children. Children.

[16] Desideri L., Pérez-Fuster P., Herrera G. (2021). Information and Communication Technologies to Support Early Screening of Autism Spectrum Disorder: A Systematic Review. Children.

[17] Muratori P., Milone A., Brovedani P., Levantini V., Melli G., Pisano S., Valente E., Thomaes S., Masi G. (2018). Narcissistic traits and self-esteem in children: Results from a community and a clinical sample of patients with oppositional defiant disorder. J. Affect. Disord..

[18] Castro E., Cotov M., Brovedani P., Coppola G., Meoni T., Papini M., Terlizzi T., Vernucci C., Pecini C., Muratori P. (2020). Associations between Learning and Behavioral Difficulties in Second-Grade Children. Children.

[19] Tobia V., Marzocchi G.M. (2018). The Strengths and Difficulties Questionnaire-Parents for Italian School-Aged Children: Psychometric Properties and Norms. Child Psychiatry Hum. Dev..

[20] Tobia V., Gabriele M.A., Marzocchi G.M. (2013). The Italian Version of the Strengths and Difficulties Questionnaire (SDQ)—Teacher. J. Psychoeduc. Assess..

[21] National Collaborating Centre for Mental Health (2005). Depression in children and young people: Identification and management in primary, community and secondary care. Database of Abstracts of Reviews of Effects (DARE): Quality-Assessed Reviews.

[22] Caro-Cañizares I., Serrano-Drozdowskyj E., Pfang B., Baca-García E., Carballo J.J. (2017). SDQ Dysregulation Profile and Its Relation to the Severity of Psychopathology and Psychosocial Functioning in a Sample of Children and Adolescents with ADHD. J. Atten. Disord..

[23] Kunze B., Wang B., Isensee C., Schlack R., Ravens-Sieberer U., Klasen F., Rothenberger A., Becker A., the BELLA-Study Group (2018). Gender associated developmental trajectories of SDQ-dysregulation profile and its predictors in children. Psychol. Med..

[24] Kivisto K.L., Welsh D.P., Darling N., Culpepper C.L. (2015). Family enmeshment, adolescent emotional dysregulation, and the moderating role of gender. J. Fam. Psychol..

[25] Goodman A., Lamping D.L., Ploubidis G.B. (2010). When to Use Broader Internalising and Externalising Subscales Instead of the Hypothesised Five Subscales on the Strengths and Difficulties Questionnaire (SDQ): Data from British Parents, Teachers and Children. J. Abnorm. Child Psychol..

[26] Guérin F., Marsh H.W., Famose J.-P. (2004). Generalizability of the PSDQ and Its Relationship to Physical Fitness: The European French Connection. J. Sport Exerc. Psychol..

[27] Dölitzsch C., Kölch M., Fegert J.M., Schmeck K., Schmid M. (2016). Ability of the Child Behavior Checklist-Dysregulation Profile and the Youth Self Report-Dysregulation Profile to identify serious psychopathology and association with correlated problems in high-risk children and adolescents. J. Affect. Disord..

[28] Kavanagh K., Hops H. (1994). Good Girls? Bad Boys? Gender and Development as Contexts for Diagnosis and Treatment. Adv. Clin. Child Psychol..

[29] Begeer S., Mandell D., Wijnker-Holmes B., Venderbosch S., Rem D., Stekelenburg F., Koot H.M. (2013). Sex Differences in the Timing of Identification among Children and Adults with Autism Spectrum Disorders. J. Autism Dev. Disord..

[30] Muratori P., Bertacchi I., Catone G., Mannucci F., Nocentini A., Pisano S., Lochman J.E. (2020). Coping Power Universal for middle school students: The first efficacy study. J. Adolesc..

[31] Masi G., Manfredi A., Nieri G., Muratori P., Pfanner C., Milone A. (2017). A Naturalistic Comparison of Methylphenidate and Risperidone Monotherapy in Drug-Naive Youth with Attention-Deficit/Hyperactivity Disorder Comorbid with Oppositional Defiant Disorder and Aggression. J. Clin. Psychopharmacol..

[32] Virtanen T., Vasalampi K., Torppa M., Lerkkanen M.-K., Nurmi J.-E. (2019). Changes in students’ psychological well-being during transition from primary school to lower secondary school: A person-centered approach. Learn. Individ. Differ..

